# Correlates of Objectively Measured Sitting Time in South Korean Adults: 2014–2015 Korea National Health and Nutrition Examination Survey

**DOI:** 10.3389/fpubh.2022.846542

**Published:** 2022-05-10

**Authors:** Hyo Lee, Miyoung Lee

**Affiliations:** ^1^Department of Sport and Health Promotion, Sangmyung University, Seoul, South Korea; ^2^Department of Sport, Health, and Rehabilitation, Kookmin University, Seoul, South Korea

**Keywords:** sedentary lifestyle, prolonged sitting, breaks from sitting, KNHANES, national health surveillance, accelerometer, physical activity monitor

## Abstract

The purpose of this study was to investigate daily amounts of time spent sitting and frequency of breaks from sitting and to identify their sociodemographic, environmental, and health behavioral correlates for Korean adults (age = 19–65). This study analyzed accelerometer subdata from the 2014–2015 Korea National Health and Nutrition Examination Survey (*n* = 1,768). Ordinary least squares regression models stratified by weekday and weekend were tested to identify correlates of time spent sitting and number of sitting breaks. The average daily amounts of sitting time during weekdays and weekends were 500.63 min (95% confidence interval [CI] = 495.20–506.06) and 488.10 min (95% CI = 481.72–494.49), respectively. On weekdays and weekends, the average numbers of breaks from sitting per hour were 6.62 (95% CI = 6.57–6.68) and 6.60 (95% CI = 6.54–6.66), respectively. The participants with the greatest daily sitting time tended to be male, middle-aged, never married, office workers, and residents of a metropolis; tended to have a high school educational level or higher; and had never smoked, were underweight, were physically inactive, and slept <6 h a day. Fewer breaks from sitting was associated with being male, never married, middle-aged, an office worker, an apartment resident, never having smoked, and underweight. Higher education level and physical inactivity were associated with more frequent breaks from sitting. To reduce sedentary behavior, this study helps identify at-risk populations and their characteristics. Future studies should incorporate longitudinal data and measure domain-specific sedentary behavior.

## Introduction

The benefits of physical activity are well-documented (1). Research has also shown that excessive and prolonged sitting time, independent of the level of physical activity, is associated with premature death, cardiovascular disease, type 2 diabetes, and cancer (2, 3). According to the updated physical activity and sitting time guidelines from the World Health Organization (WHO), sedentary behaviors should be replaced with physical activity of any intensity, which includes light-intensity physical activity, and people should perform more than the recommended levels of moderate- to vigorous-intensity physical activity (MVPA) to reduce the detrimental effects of excessive sedentary behavior (4).

In Korea, as in other developed countries, physical inactivity and excessive sitting time are prevalent, despite well-known risks. According to the Korea Disease Control and Prevention Agency (5), the estimated proportion of the population that adheres to the WHO guidelines on aerobic physical activity decreased from 58.3% in 2014 to 47.8% in 2019, when MVPA was measured with the Global Physical Activity Questionnaire (GPAQ). Furthermore, the sitting time (i.e., average number of minutes spent sitting on a typical day, measured with a single-item questionnaire) increased from 469 min in 2014 to 512 min in 2018.

To reduce sedentary behavior, the factors associated with excessive sitting must be understood so that at-risk populations can be identified, and intervention programs can be planned. Sedentary behaviors, as much as physical activity, have complex causes and patterns. Research has shown that social and physical components such as socioeconomic status and built environment have direct and indirect effects on individuals' choice of active or inactive lifestyle (6, 7). In a survey of Australian adults, for example, Hadgraft et al. (8) found that in addition to health-related factors, socioeconomic status, represented by income and occupation, was associated with prolonged sitting time. Meanwhile, previous studies on European and Singaporean multiethnic samples reported that cross-cultural variation exists in relation to the amounts and correlates of sedentary and physical activity behavior (9–11). However, studies of the determinants of sedentary behaviors in the Korean population, which could allow for international comparisons, have been lacking.

Moreover, the validity evidence of recall-based self-report questionnaires as measures of sitting time are not well-established. For example, Urda et al. (12) reported that self-reported sitting time was weakly correlated with data recorded by the activPAL3 device (PAL Technologies Ltd, Glasgow, UK) and was significantly underestimated by 44 office workers. In addition, the Global Physical Activity Questionnaire, which is widely used in national studies, has repeatedly demonstrated insufficient validity as a measure of sedentary behavior and light-intensity physical activity (13).

In summary, a study addressing sedentary behaviors in Koreans using objective physical activity monitors seems timely. We therefore investigated correlates of sitting time, measured with accelerometers, in Korean adults. Using the physical activity monitoring component of the Korea National Health and Nutrition Examination Survey (KNHANES) 2014–2015, we explored the associations between individuals' socioenvironmental and behavioral characteristics and their daily sitting time and frequency of breaks from sitting during weekdays and weekends.

## Methods

### Participants

We obtained physical activity monitor subdata (*N* = 1,768) from the KNHANES 2014–2015 database. This database reflects the non-institutionalized South Korean population according to a complex probability-sampling design. In that surveillance period, physical activity monitors were distributed to adults aged 19 to 65 years.

### Measures

Daily amounts of time spent sitting, number of breaks from sitting, and information about physical activity were extracted from raw data recorded by the ActiGraph GT3X+ (ActiGraph Corp, Pensacola, FL, USA), which is a physical activity monitor. To summarize the raw data, we used 1-minute epochs in an algorithm suggested by Troiano et al. (14). Cut points of ≤99, ≥2,020, and ≥5,999 counts per minute were applied to classify sitting time, moderate-intensity physical activity, and vigorous-intensity physical activity, respectively. According to minimum daily wear-time criterion for Koreans suggested by Lee and Lee (15), data were deemed valid when participants wore the device 10 or more hours per day. Average wear time was 11.87 and 11.03 h per day during weekdays and weekends, respectively. All missing values, including day-level data from the physical activity monitor, were imputed according to multivariate normal distribution to generate 20 complete datasets (16, 17).

Sociodemographic variables included in this study were age, marital status, household income, education, type of occupation, hours of work per week, type of housing, and area of residence. Health behavioral variables were cigarette smoking, alcohol drinking, body mass index (BMI), adherence to aerobic physical activity recommendation, and hours of sleep. All variables except for age, BMI, and physical activity adherence were self-reported. Specific classification criteria are listed the far-left column of Table 1.

**Table 1:** Average sitting time per day and breaks from sitting per hour on weekdays and weekends.

	**Sitting time per day (minutes)^a^**		**Sitting breaks per hour^b^**	
	**Weekdays**	**Weekends**	**Weekdays**	**Weekends**
Overall	500.63 (495.20 to 506.06)	488.10 (481.72 to 494.49)	6.62 (6.57 to 6.68)	6.60 (6.54 to 6.66)
Sex				
Male	504.49 (494.84 to 514.15)	489.20 (477.57 to 500.83)	6.35 (6.24 to 6.46)	6.35 (6.24 to 6.46)
Female	498.32 (492.04 to 504.59)	487.45 (479.94 to 494.95)	6.75 (6.67 to 6.82)	6.75 (6.67 to 6.82)
Age group				
10–29 years	530.35 (518.14 to 542.56)	510.39 (496.51 to 524.26)	6.30 (6.17 to 6.44)	6.34 (6.18 to 6.50)
30–29 years	493.15 (481.82 to 504.48)	483.09 (469.41 to 496.77)	6.80 (6.69 to 6.91)	6.77 (6.64 to 6.91)
40–49 years	512.64 (501.69 to 523.59)	495.70 (483.05 to 508.35)	6.72 (6.61 to 6.82)	6.69 (6.57 to 6.81)
50–59 years	485.16 (474.78 to 495.55)	476.25 (464.30 to 488.21)	6.68 (6.58 to 6.78)	6.62 (6.50 to 6.75)
60–65 years	471.11 (454.59 to 487.63)	468.49 (449.66 to 487.32)	6.58 (6.42 to 6.74)	6.48 (6.29 to 6.68)
Marital status				
Never married	535.73 (524.46 to 547.01)	518.54 (505.06 to 532.02)	6.18 (6.07 to 6.30)	6.22 (6.07 to 6.36)
Married or living with partner	489.71 (483.43 to 496.00)	478.05 (471.00 to 485.10)	6.77 (6.71 to 6.83)	6.72 (6.65 to 6.79)
Divorced, separated, or widowed	489.23 (466.24 to 512.22)	484.34 (457.99 to 510.69)	6.71 (6.50 to 6.93)	6.70 (6.44 to 6.96)
Household income				
≤ 25% (lowest)	490.77 (469.69 to 511.85)	478.59 (454.08 to 503.11)	6.42 (6.24 to 6.61)	6.42 (6.18 to 6.65)
25% < to ≤50%	480.54 (469.42 to 491.67)	475.13 (462.15 to 488.10)	6.63 (6.52 to 6.75)	6.58 (6.45 to 6.70)
50% < to ≤75%	496.63 (488.01 to 505.26)	488.10 (477.76 to 498.45)	6.67 (6.58 to 6.77)	6.63 (6.53 to 6.74)
≥75% (highest)	522.57 (513.59 to 531.55)	500.39 (490.39 to 510.39)	6.63 (6.54 to 6.72)	6.63 (6.52 to 6.73)
Educational attainment				
Middle school or less	444.61 (431.45 to 457.77)	441.20 (425.75 to 456.64)	6.62 (6.49 to 6.74)	6.51 (6.37 to 6.66)
High school	497.96 (489.48 to 506.44)	488.81 (479.62 to 498.00)	6.64 (6.55 to 6.72)	6.64 (6.54 to 6.74)
College or more	527.74 (520.27 to 535.21)	507.83 (498.36 to 517.30)	6.63 (6.54 to 6.71)	6.60 (6.50 to 6.70)
Occupation				
Office worker	539.83 (531.03 to 548.63)	519.95 (508.39 to 531.51)	6.56 (6.46 to 6.66)	6.49 (6.38 to 6.61)
Worker in a non-office setting	463.41 (453.87 to 472.95)	455.39 (444.40 to 466.39)	6.73 (6.63 to 6.82)	6.63 (6.53 to 6.74)
Economically inactive	507.97 (499.72 to 516.21)	496.66 (487.26 to 506.07)	6.58 (6.49 to 6.67)	6.65 (6.54 to 6.75)
Working hours per week				
<15 h	505.94 (498.22 to 513.67)	496.57 (488.01 to 505.12)	6.57 (6.49 to 6.65)	6.62 (6.52 to 6.71)
15≤ to <30 h	471.13 (452.96 to 489.31)	470.31 (448.67 to 491.95)	6.53 (6.35 to 6.72)	6.60 (6.39 to 6.81)
30≤ to <40 h	491.99 (474.39 to 509.58)	486.41 (463.64 to 509.18)	6.76 (6.59 to 6.93)	6.62 (6.41 to 6.83)
40≤ to <52 h*^c^*	511.81 (500.77 to 522.86)	489.19 (476.70 to 501.68)	6.61 (6.50 to 6.72)	6.57 (6.44 to 6.70)
≥52 h^d^	491.35 (476.15 to 506.56)	476.00 (459.02 to 492.98)	6.78 (6.63 to 6.92)	6.59 (6.42 to 6.75)
Type of housing				
Detached house	483.62 (474.20 to 493.04)	473.09 (461.79 to 484.39)	6.54 (6.44 to 6.64)	6.49 (6.38 to 6.60)
Apartment (condominium)	510.96 (503.85 to 518.07)	496.44 (488.35 to 504.54)	6.69 (6.62 to 6.76)	6.68 (6.59 to 6.76)
Other (e.g., multiplex, studio)	501.46 (486.76 to 516.17)	492.01 (474.38 to 509.65)	6.61 (6.46 to 6.76)	6.57 (6.39 to 6.75)
City size				
Metropolis	513.14 (506.48 to 519.80)	498.58 (490.91 to 506.25)	6.60 (6.53 to 6.66)	6.58 (6.51 to 6.66)
Urban	488.70 (477.22 to 500.18)	483.01 (469.69 to 496.34)	6.76 (6.64 to 6.88)	6.73 (6.59 to 6.88)
Rural	465.54 (451.80 to 479.29)	453.52 (437.75 to 469.29)	6.61 (6.49 to 6.74)	6.52 (6.37 to 6.67)
Smoking status				
Never smoked	505.20 (498.91 to 511.48)	491.30 (483.91 to 498.69)	6.68 (6.62 to 6.74)	6.66 (6.58 to 6.73)
Stopped smoking	486.02 (472.71 to 499.33)	478.81 (463.97 to 493.65)	6.47 (6.34 to 6.60)	6.42 (6.27 to 6.56)
Currently smoking	497.87 (483.02 to 512.72)	485.06 (467.30 to 502.81)	6.59 (6.44 to 6.74)	6.57 (6.38 to 6.75)
Drinking alcohol				
Never drank in the past year	496.18 (485.17 to 507.19)	485.65 (472.91 to 498.39)	6.70 (6.60 to 6.81)	6.62 (6.49 to 6.75)
Once or less a month	504.19 (495.22 to 513.17)	494.00 (483.61 to 504.39)	6.62 (6.53 to 6.71)	6.65 (6.55 to 6.76)
Once or less a week	509.95 (499.18 to 520.72)	493.93 (481.63 to 506.24)	6.58 (6.47 to 6.69)	6.54 (6.42 to 6.67)
Twice or more a week	487.07 (473.80 to 500.35)	472.68 (457.06 to 488.30)	6.62 (6.49 to 6.75)	6.56 (6.41 to 6.71)
Body Mass Index (kg/m^2^)*^e^*				
<18.5	538.73 (513.79 to 563.67)	515.76 (489.42 to 542.11)	6.37 (6.11 to 6.63)	6.47 (6.16 to 6.78)
18.5≤ to <25	505.45 (498.76 to 512.12)	490.91 (483.39 to 498.43)	6.63 (6.57 to 6.70)	6.61 (6.53 to 6.69)
25≤ to <30	484.99 (473.80 to 496.19)	479.60 (466.76 to 492.44)	6.66 (6.55 to 6.76)	6.61 (6.49 to 6.73)
≥30	482.85 (453.04 to 512.65)	469.58 (438.05 to 501.11)	6.64 (6.38 to 6.91)	6.53 (6.27 to 6.78)
Physical activity adherence				
Not adhering	519.42 (510.58 to 528.26)	510.60 (499.66 to 521.54)	6.90 (6.81 to 7.00)	6.86 (6.75 to 6.97)
Adhering^*f*^	490.55 (483.71 to 497.40)	476.02 (466.80 to 485.25)	6.48 (6.42 to 6.54)	6.46 (6.38 to 6.54)
Sleeping hours per day				
≤6 h^g^	510.26 (501.60 to 518.93)	493.63 (483.54 to 503.71)	6.59 (6.51 to 6.68)	6.59 (6.49 to 6.69)
7 h^h^	500.91 (491.33 to 510.49)	489.79 (479.33 to 500.24)	6.67 (6.56 to 6.77)	6.63 (6.51 to 6.74)
≥8 h^i^	488.42 (478.61 to 498.22)	479.66 (468.37 to 490.94)	6.64 (6.54 to 6.73)	6.58 (6.47 to 6.68)

a *Average minutes per day with 95% confidence interval*.

b *Average number of breaks per hour during physical activity monitor wear time (95% confidence interval)*.

c *According to Labor Standards Act of South Korea, 40 h per week is the maximum work hours for employees*.

d *According to Labor Standards Act of South Korea in special cases, 52 h per week is the maximum work hours for employees*.

e *The lower BMI limits of normal to overweight, class 1 obesity, and class 2–3 obesity are 18.5, 25, and 30 kg/m^2^, respectively (18)*.

f *150 min per week of moderate-intensity physical activity, 75 min per week of vigorous physical activity, or an equivalent combination of moderate-intensity to vigorous physical activity*.

g *First quartile*.

h *Second quartile*.

i *Third quartile*.

### Statistical Analyses

Average daily time spent sitting and sitting breaks per hour on weekdays and weekends were estimated. Ordinary least squares regression models were fitted to the data to test sociodemographic and behavioral correlates of sitting time per day and sitting breaks per hour. All estimates were pooled from the multiple-imputation datasets. To process data from the physical activity monitors, we used SAS 9.2 (IBM Corporation, Armonk, NY, USA). Stata 12.0 (StataCorp LLC, College Station, TX, USA) was used to perform all other analyses.

## Results

Average daily sitting time (minutes) and number of breaks from sitting (per hour) are listed in Table 1. On average, participants sat for 500.63 min on weekdays (95% confidence interval [CI] = 495.20–506.06) and 488.10 min on weekends (95% CI = 481.72–494.49). The average numbers of breaks from sitting per hour were 6.62 times on weekdays (95% CI = 6.57–6.68) and 6.60 times on weekends (95% CI = 6.54–6.66), respectively.

### Correlates of Sitting Time

Table 2 shows data from unadjusted and adjusted least squares regression models used to test correlates of sitting time. According to the adjusted model, longer sitting time during the week was associated significantly more with being male than with being female (*B* = 22.67, *p* < 0.01); with never being married than with either being married or living with a partner (*B* = 41.58, *p* < 0.001); with graduation from high school (*B* = 34.12, *p* < 0.001) or higher education levels (*B* = 45.53, *p* < 0.001) than with less than a high-school education; with being an office worker than working in a non-office setting (*B* = 52.30, *p* < 0.001); with living in a metropolis than living in urban areas (*B* = 17.89, *p* < 0.01) and rural areas (*B* = 27.38, *p* < 0.001); with being underweight than class 1 obesity (*B* = 27.01, *p* < 0.05) or class 2–3 obesity (*B* = 33.40, *p* < 0.05); with never having smoked than having quit smoking (*B* = 17.12, *p* < 0.05); with being physically inactive than being active (*B* = 36.17, *p* < 0.001); and with sleeping 6 h or less a night than sleeping 7 h (*B* = 12.00, *p* < 0.05) or 8 h or more a night (*B* = 21.41, *p* < 0.01). The adjusted model also showed that during weekends, people aged 40–49 (*B* = 28.43, *p* < 0.05) and 50–59 years (*B* = 30.66, *p* < 0.05) sat significantly longer than those aged 19–29 years; those who had never married sat significantly longer than did those who were married or living with a partner (*B* = 47.33, *p* < 0.001); high school graduates (*B* = 33.91, *p* < 0.001) and those with more than college education attainment (*B* = 36.93, *p* < 0.01) sat significantly longer than those with less than a high-school education; office workers sat significantly longer than did those who worked in non-office settings (*B* = 49.15, *p* < 0.001); and those who lived in a metropolis sat significantly longer than did those living in a rural area (*B* = 29.54, *p* < 0.01). Among health behaviors, only physical activity (physically inactive vs. active) was significantly associated with weekend sitting time (*B* = 41.99, *p* < 0.001).

**Table 2:** Ordinary least squares regression models testing correlates of daily sitting time.

	**Weekdays**				**Weekend**			
	**Unadjusted models**		**Adjusted model**		**Unadjusted models**		**Adjusted model**	
	***B* (95% CI)**	***p***	***B* (95% CI)**	***p***	***B* (95% CI)**	***p***	***B* (95% CI)**	***p***
Sex								
Male	Reference group							
Female	−6.18 (−17.14 to 4.78)	0.269	−22.67 (−36.32 to −9.03)	0.001	−1.75 (−15.32 to 11.82)	0.080	−16.20 (−33.73 to 1.34)	0.070
Age group								
10–29 years	Reference group							
30–29 years	−37.20 (−54.24 to −20.16)	<0.001	−13.76 (−33.7 to 6.19)	0.176	−27.3 (−46.75 to −7.84)	0.006	1.34 (−22.74 to 25.42)	0.913
40–49 years	−17.71 (−34.59 to −0.83)	0.040	20.72 (−0.85 to 42.29)	0.060	−14.69 (−32.58 to 3.21)	0.108	28.43 (3.00 to 53.87)	0.029
50–59 years	−45.19 (−61.41 to −28.96)	<0.001	18.21 (−4.28 to 40.70)	0.112	−34.13 (−52.01 to −16.25)	<0.001	30.66 (4.08 to 57.24)	0.024
60–65 years	−59.24 (−80.06 to −38.41)	<0.001	14.85 (−11.43 to 41.14)	0.268	−41.9 (−65.73 to −18.06)	0.001	31.06 (0.05 to 62.06)	0.050
Marital status								
Never married	Reference group							
Married or living with partner	−46.02 (−59.17 to −32.88)	<0.001	−41.58 (−59.89 to −23.27)	<0.001	−40.49 (−55.12 to −25.86)	<0.001	−47.33 (−69.21 to −25.46)	<0.001
Divorced, separated, or widowed	−46.50 (−70.66 to −22.35)	<0.001	−25.26 (−52.44 to 1.93)	0.069	−34.20 (−60.42 to −7.98)	0.011	−28.74 (−60.44 to 2.96)	0.075
Household income								
≤ 25% (lowest)	Reference group							
25% < to ≤50%	−10.23 (−31.90 to 11.45)	0.355	−14.71 (−35.46 to 6.04)	0.164	−3.46 (−28.04 to 21.11)	0.782	−5.66 (−29.87 to 18.54)	0.646
50% < to ≤75%	5.86 (−14.88 to 26.61)	0.579	−9.85 (−30.17 to 10.47)	0.342	9.51 (−14.73 to 33.74)	0.441	−0.66 (−24.96 to 23.64)	0.957
≥75% (highest)	31.80 (10.53 to 53.08)	0.003	5.47 (−15.84 to 26.78)	0.614	21.79 (−1.79 to 45.38)	0.070	4.26 (−20.15 to 28.66)	0.732
Educational attainment								
Middle school or less	Reference group							
High school	53.35 (38.49 to 68.21)	<0.001	34.12 (17.83 to 50.41)	<0.001	47.61 (30.70 to 64.52)	<0.001	33.91 (15.04 to 52.78)	<0.001
College or more	83.13 (68.62 to 97.64)	<0.001	45.53 (27.4 to 63.66)	<0.001	66.63 (49.09 to 84.18)	<0.001	36.93 (15.08 to 58.78)	0.001
Occupation								
Office worker	Reference group							
Worker in a non-office setting	−76.42 (−89.15 to −63.69)	<0.001	−52.3 (−66.56 to −38.04)	<0.001	−64.55 (−79.49 to −49.62)	<0.001	−49.15 (−66.55 to −31.76)	<0.001
Economically inactive	−31.86 (−44.88 to −18.85)	<0.001	−11.87 (−34.85 to 11.11)	0.311	−23.29 (−39.00 to −7.57)	0.004	−27.71 (−56.44 to 1.01)	0.059
Working hours per week								
<15 h	Reference group							
15≤ to <30 h	−34.81 (−54.89 to −14.73)	0.001	−5.15 (−32.84 to 22.53)	0.715	−26.26 (−48.91 to −3.60)	0.023	−12.35 (−44.52 to 19.81)	0.450
30≤ to <40 h	−13.96 (−33.15 to 5.24)	0.154	8.14 (−17.92 to 34.20)	0.540	−10.16 (−32.71 to 12.40)	0.377	−2.19 (−35.62 to 31.25)	0.897
40≤ to <52 h^*a*^	5.87 (−7.95 to 19.69)	0.405	10.06 (−13.56 to 33.68)	0.403	−7.37 (−22.89 to 8.14)	0.351	−16.02 (−43.46 to 11.43)	0.252
≥52 h^b^	−14.59 (−30.14 to 0.96)	0.066	15.68 (−8.95 to 40.31)	0.212	−20.57 (−38.38 to −2.75)	0.024	−6.33 (−35.71 to 23.05)	0.672
Type of housing								
Detached house	Reference group							
Apartment (condominium)	27.34 (15.91 to 38.77)	<0.001	8.65 (−2.56 to 19.85)	0.130	23.36 (9.85 to 36.86)	0.001	9.39 (−3.81 to 22.60)	0.163
Other (e.g., multiplex, studio)	17.84 (0.71 to 34.97)	0.041	7.96 (−8.07 to 23.99)	0.330	18.92 (−1.26 to 39.10)	0.066	10.08 (−9.31 to 29.47)	0.307
City size								
Metropolis	Reference group							
Urban	−24.44 (−38.28 to −10.61)	0.001	−17.89 (−30.99 to −4.79)	0.008	−15.57 (−31.18 to 0.05)	0.051	−10.50 (−25.71 to 4.70)	0.175
Rural	−47.59 (−61.83 to −33.35)	<0.001	−27.73 (−41.42 to −14.03)	<0.001	−45.06 (−61.72 to −28.41)	<0.001	−29.54 (−46.24 to −12.84)	0.001
Smoking status								
Never smoked	Reference group							
Stopped smoking	−19.18 (−33.21 to −5.15)	0.007	−17.12 (−32.63 to −1.61)	0.031	−12.49 (−28.95 to 3.98)	0.137	−5.21 (−24.45 to 14.04)	0.595
Currently smoking	−7.32 (−22.47 to 7.82)	0.343	−6.35 (−23.34 to 10.65)	0.464	−6.24 (−23.95 to 11.46)	0.489	0.22 (−19.67 to 20.12)	0.982
Drinking alcohol								
Never drank in the past year	Reference group							
Once or less a month	8.01 (−6.43 to 22.45)	0.276	−1.79 (−15.28 to 11.69)	0.794	8.35 (−8.05 to 24.75)	0.318	1.49 (−14.35 to 17.33)	0.853
Once or less a week	13.77 (−1.51 to 29.06)	0.077	−2.83 (−17.61 to 11.95)	0.707	8.28 (−9.53 to 26.09)	0.361	−4.19 (−22.02 to 13.65)	0.645
Twice or more a week	−9.11 (−26.15 to 7.930)	0.294	−13.51 (−30.77 to 3.74)	0.124	−12.97 (−32.88 to 6.94)	0.201	−16.25 (−36.03 to 3.53)	0.107
Body Mass Index (kg/m^2^)^*c*^								
<18.5	Reference group							
18.5≤ to <25	−33.29 (−59.59 to −6.99)	0.013	−18.69 (−43.79 to 6.41)	0.144	−24.85 (−54.77 to 5.07)	0.103	−11.80 (−39.97 to 16.37)	0.411
25≤ to <30	−53.74 (−81.26 to −26.22)	<0.001	−30.26 (−56.53 to −3.99)	0.024	−36.16 (−67.23 to −5.10)	0.023	−15.09 (−44.87 to 14.70)	0.320
≥30	−55.89 (−90.71 to −21.06)	0.002	−36.62 (−69.72 to −3.52)	0.030	−46.18 (−87.24 to −5.12)	0.028	−27.87 (−65.86 to 10.13)	0.150
Physical activity adherence								
Not adhering	Reference group							
Adhering^*d*^	−28.87 (−40.3 to −17.43)	<0.001	−36.17 (−47 to −25.33)	<0.001	−34.58 (−50.36 to −18.79)	<0.001	−41.99 (−56.63 to −27.35)	<0.001
Sleeping hours per day								
≤ 6 h^e^	Reference group							
7 h^f^	−9.35 (−22.16 to 3.46)	0.152	−12 (−23.72 to −0.27)	0.045	−3.84 (−18.13 to 10.45)	0.598	−5.84 (−19.85 to 8.17)	0.413
≥8 h^g^	−21.85 (−34.97 to −8.72)	0.001	−21.41 (−33.64 to −9.18)	0.001	−13.97 (−28.66 to 0.72)	0.062	−14.67 (−29.01 to −0.32)	0.045

*The adjusted model is the multiple regression model that includes all independent variables as covariates*.

a *According to Labor Standards Act of South Korea, 40 h per week is the maximum work hours for employees*.

b *According to Labor Standards Act of South Korea in special cases, 52 h per week is the maximum work hours for employees*.

c *The lower BMI limits of normal to overweight, class 1 obesity, and class 2–3 obesity are 18.5, 25, and 30 kg/m^2^, respectively (18)*.

d *150 min per week of moderate-intensity physical activity, 75 min per week of vigorous physical activity, or an equivalent combination of moderate-intensity to vigorous physical activity*.

e *First quartile*.

f *Second quartile*.

g *Third quartile*.

### Correlates of (Fewer) Breaks From Sitting

Results from unadjusted and adjusted least squares regression models to test correlates of breaks from sitting are listed in Table 3. The adjusted model showed that during weekdays, the following participants tended have fewer sitting breaks: those who were male, in comparison with those who were female (*B* = −0.54, *p* < 0.001); those aged 40–49 years (*B* = −0.27, *p* < 0.05) and 50–59 years (*B* = −0.26, *p* < 0.05), in comparison with those aged 19–29; those never married, in comparison with those married or living with a partner (*B* = −0.66, *p* < 0.001) and with those who were divorced, separated, or widowed (*B*= −0.58, *p* < 0.001); those who worked in an office, in comparison with those who worked in non-office settings (*B* = −0.16, *p* < 0.05); those who lived in a detached home, in comparison with those who lived in apartments or condominiums (*B* = −0.14, *p* < 0.05); those who had never smoked, in comparison with those currently smoking (*B* = −0.21, *p* < 0.05); those who were underweight, in comparison with those who were normal to overweight (*B* = −0.27, *p* < 0.05) and obese (class 1; *B* = −0.32, *p* < 0.05); and those who were physically active, in comparison with those who were inactive (*B* = −0.33, *p* < 0.001). During weekends, the following participants took less breaks from sitting: individuals who were male, in comparison with those who were female (*B* = −0.43, *p* < 0.001); those who had never married, in comparison with both those who were married or living with a partner (*B* = −0.62, *p* < 0.001) and those who were divorced, separated, or widowed (*B* = −0.61, *p* < 0.001); those with a middle school education or less, in comparison with high school graduates (*B* = −0.20, *p* < 0.05); those who worked in an office, in comparison with those who worked in non-office settings (*B* = −0.19, *p* < 0.05); and those who were physically active, in comparison with those who were inactive (*B* = −0.32, *p* < 0.001).

**Table 3:** Ordinary least squares regression models testing correlates of breaks from sitting per hour.

	**Weekdays**				**Weekend**			
	**Unadjusted models**		**Adjusted model**		**Unadjusted models**		**Adjusted model**	
	***B* (95% CI)**	***p***	***B* (95% CI)**	***p***	***B* (95% CI)**	***p***	***B* (95% CI)**	***p***
Sex								
Male	Reference group							
Female	0.42 (0.31 to 0.53)	<0.001	0.54 (0.40 to 0.68)	<0.001	0.40 (0.26 to 0.53)	<0.001	0.43 (0.26 to 0.60)	<0.001
Age group								
10–29 years	Reference group							
30–29 years	0.50 (0.33 to 0.67)	<0.001	−0.09 (−0.30 to 0.13)	0.434	0.43 (0.22 to 0.65)	<0.001	−0.07 (−0.32 to 0.18)	0.585
40–49 years	0.42 (0.25 to 0.58)	<0.001	−0.27 (−0.50 to −0.05)	0.019	0.35 (0.15 to 0.54)	0.001	−0.25 (−0.52 to 0.01)	0.060
50–59 years	0.37 (0.22 to 0.53)	<0.001	−0.26 (−0.50 to −0.03)	0.030	0.28 (0.09 to 0.48)	0.005	−0.27 (−0.56 to 0.01)	0.063
60–65 years	0.28 (0.07 to 0.48)	0.008	−0.24 (−0.52 to 0.03)	0.085	0.14 (−0.12 to 0.40)	0.284	−0.31 (−0.64 to 0.02)	0.070
Marital status								
Never married	Reference group							
Married or living with partner	0.59 (0.46 to 0.72)	<0.001	0.66 (0.47 to 0.85)	<0.001	0.50 (0.33 to 0.67)	<0.001	0.62 (0.39 to 0.86)	<0.001
Divorced, separated, or widowed	0.53 (0.30 to 0.76)	<0.001	0.58 (0.29 to 0.86)	<0.001	0.48 (0.21 to 0.75)	0.001	0.61 (0.27 to 0.94)	<0.001
Household income								
≤25% (lowest)	Reference group							
25% < to ≤50%	0.21 (−0.01 to 0.44)	0.064	0.07 (−0.15 to 0.29)	0.539	0.16 (−0.10 to 0.42)	0.233	0.03 (−0.23 to 0.28)	0.831
50% < to ≤75%	0.25 (0.03 to 0.47)	0.024	0.09 (−0.12 to 0.31)	0.405	0.22 (−0.04 to 0.47)	0.101	0.07 (−0.20 to 0.34)	0.618
≥75% (highest)	0.20 (−0.01 to 0.42)	0.063	0.07 (−0.15 to 0.29)	0.539	0.21 (−0.05 to 0.46)	0.112	0.08 (−0.19 to 0.35)	0.563
Educational attainment								
Middle school or less	Reference group							
High school	0.02 (−0.13 to 0.17)	0.795	0.15 (−0.02 to 0.31)	0.081	0.12 (−0.05 to 0.30)	0.173	0.20 (0.00 to 0.39)	0.046
College or more	0.01 (−0.14 to 0.17)	0.879	0.12 (−0.07 to 0.31)	0.219	0.08 (−0.09 to 0.26)	0.347	0.14 (−0.08 to 0.36)	0.206
Occupation								
Office worker	Reference group							
Worker in a non-office setting	0.16 (0.03 to 0.30)	0.017	0.16 (0.01 to 0.31)	0.038	0.14 (−0.01 to 0.29)	0.069	0.19 (0.02 to 0.36)	0.032
Economically inactive	0.02 (−0.12 to 0.15)	0.811	0 (−0.24 to 0.24)	0.984	0.16 (−0.01 to 0.32)	0.063	0.20 (−0.08 to 0.49)	0.166
Working hours per week								
<15 h	Reference group							
15≤ to <30 h	−0.04 (−0.24 to 0.16)	0.696	−0.04 (−0.32 to 0.24)	0.788	−0.02 (−0.25 to 0.22)	0.882	0.14 (−0.18 to 0.46)	0.389
30≤ to <40 h	0.19 (−0.01 to 0.38)	0.057	0.12 (−0.15 to 0.39)	0.400	0.00 (−0.23 to 0.24)	0.968	0.08 (−0.23 to 0.4)	0.604
40≤ to <52 h^*a*^	0.03 (−0.10 to 0.17)	0.632	0.12 (−0.12 to 0.36)	0.323	−0.04 (−0.21 to 0.13)	0.623	0.17 (−0.11 to 0.46)	0.224
≥52 h^b^	0.20 (0.05 to 0.36)	0.010	0.25 (−0.01 to 0.51)	0.057	−0.03 (−0.21 to 0.16)	0.760	0.14 (−0.16 to 0.43)	0.359
Type of housing								
Detached house	Reference group							
Apartment (condominium)	0.15 (0.03 to 0.27)	0.013	0.14 (0.01 to 0.26)	0.028	0.19 (0.05 to 0.32)	0.007	0.13 (−0.01 to 0.27)	0.076
Other (e.g., multiplex, studio)	0.07 (−0.11 to 0.26)	0.437	0.02 (−0.15 to 0.2)	0.789	0.08 (−0.13 to 0.30)	0.455	0.01 (−0.2 to 0.22)	0.900
City size								
Metropolis	Reference group							
Urban	0.16 (0.02 to 0.30)	0.027	0.11 (−0.03 to 0.25)	0.111	0.15 (−0.02 to 0.32)	0.075	0.10 (−0.06 to 0.27)	0.221
Rural	0.02 (−0.13 to 0.16)	0.832	−0.08 (−0.23 to 0.06)	0.257	−0.06 (−0.23 to 0.11)	0.478	−0.12 (−0.29 to 0.05)	0.173
Smoking status								
Never smoked	Reference group							
Stopped smoking	−0.21 (−0.35 to −0.07)	0.004	0.05 (−0.11 to 0.21)	0.552	−0.24 (−0.40 to −0.08)	0.004	0.01 (−0.17 to 0.19)	0.917
Currently smoking	−0.09 (−0.24 to 0.07)	0.260	0.21 (0.02 to 0.39)	0.026	−0.09 (−0.29 to 0.11)	0.377	0.20 (−0.03 to 0.43)	0.087
Drinking alcohol								
Never drank in the past year	Reference group							
Once or less a month	−0.08 (−0.23 to 0.06)	0.276	−0.05 (−0.19 to 0.09)	0.447	0.03 (−0.14 to 0.20)	0.694	0.05 (−0.12 to 0.22)	0.579
Once or less a week	−0.12 (−0.28 to 0.04)	0.131	−0.01 (−0.17 to 0.15)	0.859	−0.07 (−0.26 to 0.11)	0.424	0.02 (−0.17 to 0.21)	0.835
Twice or more a week	−0.08 (−0.25 to 0.09)	0.345	0.01 (−0.16 to 0.19)	0.893	−0.06 (−0.26 to 0.14)	0.556	0.04 (−0.18 to 0.26)	0.749
Body Mass Index (kg/m^2^)^*c*^								
<18.5	Reference group							
18.5≤ to <25	0.27 (−0.01 to 0.54)	0.057	0.27 (0.01 to 0.53)	0.043	0.14 (−0.16 to 0.44)	0.365	0.16 (−0.13 to 0.46)	0.271
25≤ to <30	0.29 (0.01 to 0.57)	0.042	0.32 (0.05 to 0.59)	0.022	0.14 (−0.17 to 0.45)	0.375	0.20 (−0.11 to 0.51)	0.204
≥30	0.28 (−0.11 to 0.66)	0.164	0.35 (−0.02 to 0.72)	0.066	0.06 (−0.35 to 0.46)	0.784	0.15 (−0.24 to 0.54)	0.459
Physical activity adherence								
Not adhering	Reference group							
Adhering^*d*^	−0.42 (−0.54 to −0.31)	<0.001	−0.33 (−0.45 to −0.22)	<0.001	0.03 (−0.11 to 0.18)	0.644	−0.32 (−0.47 to −0.17)	<0.001
Sleeping hours per day								
≤6 h^e^	Reference group							
7 h^f^	0.07 (−0.06 to 0.21)	0.277	0.10 (−0.02 to 0.23)	0.106	−0.40 (−0.55 to −0.25)	<0.001	0.05 (−0.09 to 0.20)	0.465
≥8 h^g^	0.04 (−0.09 to 0.17)	0.525	0.10 (−0.03 to 0.22)	0.130			0.00 (−0.15 to 0.14)	0.980

*The adjusted model is the multiple regression model that includes all independent variables as covariates*.

a *According to Labor Standards Act of South Korea, 40 h per week is the maximum work hours for employees*.

b *According to Labor Standards Act of South Korea in special cases, 52 h per week is the maximum work hours for employees*.

c *The lower BMI limits of normal to overweight, class 1 obesity, and class 2–3 obesity are 18.5, 25, and 30 kg/m^2^, respectively (18)*.

d *150 min per week of moderate-intensity physical activity, 75 min per week of vigorous physical activity, or an equivalent combination of moderate-intensity to vigorous physical activity*.

e *First quartile*.

f *Second quartile*.

g *Third quartile*.

## Discussion

This study, to the best of our knowledge, was the first to analyze sociodemographic and health behavior correlates of objectively measured sitting time using Korean national surveillance data. In view of the growing evidence of the adverse effects of excessive sitting, it is important to identify factors associated with sedentary behavior in South Korean adults; these factors can help identify at-risk groups and inform intervention programs.

We found that Korean adults spent 500.63 min per weekday and 488.10 min per day on the weekend sitting (being sedentary). The difference between weekdays and weekends may not be meaningful; however, as participants wore the monitor substantially longer on weekdays (11.87 h) than on weekends (11.03 h). The numbers of breaks from sitting were 6.62 and 6.60 times per hour on weekdays and weekends, respectively. Among national surveillance data from other countries, those of the National Health and Nutrition Examination Survey (NHANES) 2004–2005 in the United States (19) are comparable with ours, despite the 10-year gap. The single-axis accelerometric data of 3,725 participants indicated that, on average, adults in the United States were sedentary for 478.9 min per day and took 6.54 breaks from sitting per hour in 2004 and 2005. Considering that the average amount of time that the NHANES participants wore the monitor was 14.0 h per day, Korean adults in 2014–2015 seem to have spent more time sitting than did American adults in 2004–2005. To calculate how much the Korean adults would have sat if they had worn the device for 14.0 h a day, we divided their average sitting time by wear time and then multiplied the answer by wear time recorded for the US adults (500.6 × 14.0/11.9 = 588.9 min on weekdays and 488.1 × 14.0/11.0 = 621.2 min on weekends). Meanwhile, the results from previous studies in East Asian countries were not directly comparable with ours because those studies involved different measurement modes (i.e., objective vs. subjective) (9) or different domains of sedentary behavior (e.g., all domains vs. occupational sitting time) (20).

### Correlates of Sitting Time

In the univariate (unadjusted) models, all independent variables except for sex and alcohol drinking were significantly associated with weekday sitting time. On weekends, in addition to sex and alcohol drinking, household income, smoking status, and number of hours of sleep were not significantly associated with sitting time. According to the multiple regression models, never having been married, having a high school or higher education level, being an office worker, residing in a metropolis, and physical inactivity (i.e., not adhering to MVPA guidelines) were significant risk factors for excessive sitting during both weekdays and weekends when other variables were adjusted. Being male, never having smoked, being underweight, and sleeping 6 h or less at night were the factors that were significantly and positively associated sitting time during weekdays only. People in their 40s and 50s spent significantly more time sitting during weekends than those in their 20s.

Previous studies have shown that being male, being older, being single, higher BMI, higher education, being an office worker, living in an urban or metropolitan area, smoking, drinking alcohol, shorter sleep hours, and less physical activity were associated with longer sitting time in adults (8–10, 20–25). It was reported that prolonged sitting combined with physical inactivity is associated with an increased risk of mortality (26). Our study shows that prolonged sitting is associated not only with physical inactivity but also with other unhealthy behaviors. Future studies should identify at-risk populations and the health effects of these combinations. Meanwhile, most of our findings were consistent with previous studies; the exceptions were those for smoking and BMI.

In our study, individuals who had stopped smoking sat longer on weekdays than those who had never smoked. This finding does not necessarily contradict the results of the studies previously mentioned because smoking cessation is considered as much a health-enhancing and purposeful behavior as never smoking. Further, in our study, participants who were underweight sat significantly longer on weekdays than did their counterparts who were obese. A few studies of adolescents produced findings that may be instructive in understanding these somewhat counterintuitive findings; for example, Polish adolescents who were underweight were less physically active than those of normal weight (27). Similarly, Artero et al. (28) found that physical fitness level was poorer among underweight Spanish adolescents than among their normal-weight counterparts. These findings suggest that less sedentary behavior and more physical activity can be beneficial for maintaining a healthy weight; but being underweight may also hinder an active lifestyle because of the lack of physical fitness.

An association between an independent variable and a dependent variable that is significant in an unadjusted model might not be significant in an adjusted model; for example, age was significantly correlated with weekday sitting time in our unadjusted model but not in our adjusted model. This suggests that in young people who are sedentary, their inactivity is not attributable to age (spurious association); rather, they are more likely to never have been married, to be recipients of higher education, and to be white-collar workers, among other variables. Associations between weekday sitting time and age, household income, working hours per week, and type of housing appeared spurious. Moreover, on weekends, the associations between sitting time and both working hours per week and BMI seemed spurious, too.

### Correlates of (Fewer) Breaks From Sitting

In the univariate regression models, sex, age, marital status, household income, occupation, working hours per week, type of housing, city size, smoking status, and adherence to MVPA guidelines were significantly associated with number of breaks from sitting per hour on weekdays. On weekends, among these, however, household income and occupation were not significantly correlated with the number sitting breaks. The multivariate regression models showed that participants who were male, had never married, were office workers (vs. working in non-office settings), and were physically active (i.e., adhering to MVPA guidelines) took fewer breaks from sitting than did their counterparts during both weekdays and weekends. Age in the 40s and 50s (vs. 20s), residing in apartments (vs. detached houses), never having smoked (vs. currently smoking), and being underweight (vs. obese) were risk factors for fewer sitting breaks during weekdays. Lastly, lower education attainment (completion of middle school or less vs. high school) was associated with less frequent sitting breaks during weekends.

Associations that are significant only when potential confounding factors were not accounted for can be considered spurious. Such associations were found between sitting breaks and household income, working hours per week, and city size on weekdays; and age, type of housing, and smoking status on weekends.

Because self-report questionnaires have shown limited validity evidence in measuring the number of breaks from sitting, only a handful of studies have examined its correlates using objective measures. In a study of 227 Japanese office-based workers, Kurita et al. (20) reported that being male, residing in a metropolitan area, being overweight or obese, and physical inactivity were risk factors for taking fewer breaks from sitting. Further, in a cross-sectional study of 205 Danish blue-collar workers, Gupta et al. (29) found that BMI was negatively associated with short-term sitting (<5 min) and positively associated with long-term sitting (>30 min). In a longitudinal study of 1,536 older English adults, Yerrakalva et al. (30) reported that higher BMI, more television viewing, and less physical activity were associated with a higher number of prolonged sedentary periods (fewer breaks from sitting).

These results are consistent with our findings, except those for BMI and physical activity. Rather than contradicting results of previous studies, our findings suggest that sedentary behavior may be attributed to health status, including BMI, and the opposite direction of influences may be true as well (i.e., a reciprocal relationship that engaging in less physical activity and more sedentary behavior results in poor health status such as overweight/obesity; but also being underweight that may involve lack of physical fitness may result in fewer sitting breaks). Nevertheless, it is hard to reason why physically active participants did not take as many breaks from sitting as their physically inactive counterparts. In contrast, total sitting time was shorter among physically active participants than among inactive ones. With regards to this, the readers should remind that sitting, light-intensity physical activity, and MVPA represent a continuum of movement intensity (31). Therefore, sitting less means being more physically active. Our findings suggest that having fewer sitting breaks may have different etiology compared to total sitting time. Otherwise, physically active individuals may have not broken sitting time simply because they sat less than inactive ones. Future study needs to address this issue by incorporating motivational components and different measurement approaches to sitting break (e.g., counting the number of prolonged sedentary periods of certain criterion and/or sitting break per “sitting” hour).

We also found that age in the 40s and 50s (vs. 20s), never being married, being a current smoker, and living in an apartment are risk factors for fewer sitting breaks. In Korea, middle-aged adults are the subpopulation that is economically most active but also a group whose risk for chronic diseases and premature death has increased steeply over the past 10 years (32). In addition, studies have consistently demonstrated that the physical activity adherence among people in their 40s to 60s is higher than that among people in their 30s (17, 33); thus middle-aged adults in Korea are both physically active but also the most sedentary age group.

With regard to the associations between marital status and daily sitting time and breaks from sitting, previous studies showed that unmarried women tended to have longer workplace sitting time and to watch more TV than did their married counterparts (8, 34); the reason may be that unmarried adults may have fewer family obligations than do married adults. A related finding is that a high burden of family support was associated with less sedentary behavior (10, 35). Workers who smoke presumably need to interrupt sitting more often to smoke outdoors because indoor smoking is prohibited, but our results indicate the opposite. Future studies should address how the frequency of smoking is related to taking breaks from sitting.

Living in an apartment in Korea means having many conveniences, including home automation and well-designed neighborhoods. Many new apartment complexes include high-quality sports facilities, parks, and safe walking environments. According to Lee et al. (36), an apartment complex was indeed one of the favorite places for older adults to walk.

In sum, our study results confirmed that the total amount of sitting and breaking up long-lasting sitting have different correlates, although they also have some degree of commonality. Previous research showed that short- and long-term physiological responses to too much daily sitting time and fewer breaks from sitting are interrelated but different (3, 18, 29, 37, 38). In view of these findings, these two behaviors must be differentiated to identify at-risk populations and to design intervention programs.

A strength of this study was that accelerometers were used to objectively measure total sitting time, number of breaks from sitting per hour, and MVPA. In addition, the participants of this study were recruited as part of a national surveillance system. Although the subsample of this study was not randomly selected, the KNHANES is a random and representative sample of the non-institutionalized Korean population. However, this study had some limitations. First, the KNHANES is a cross-sectional surveillance system, and so cause and effect association could not be confirmed in the current study. Second, we did not differentiate domains of sedentary behaviors, i.e., we were not able to identify whether sitting time and breaks from sitting took place at home, in the workplace, at school, during leisure time, and in other situations, which could have informed further on etiology of sedentary behaviors and domain-specific intervention programs.

## Conclusions

In summary, we found that in the period 2014–2015, South Korean adults spent 500.63 and 488.10 min a day sitting on weekdays and weekends, respectively. In addition, they took 6.62 and 6.60 breaks from sitting per hour on weekdays and weekends, respectively. The people who spent the most time sitting were male, middle-aged, and never married; had a high school or higher level of education; were office workers and residents of metropolises; never smoked; were underweight and physically inactive; and slept less than 6 h a day. The people who sat for prolonged periods (i.e., least frequent sitting breaks) group were predominantly male, never married, middle-aged, office workers, apartment residents, and had never smoked and were underweight. Higher education and physical inactivity were associated with more breaks from sitting. In future studies, investigators should consider the contexts of sedentary behaviors (e.g., work, leisure, screen time, and transportation) and use a longitudinal study design.

## Data Availability Statement

The datasets presented in this study can be found in online repositories. The names of the repository/repositories and accession number(s) can be found at: https://knhanes.kdca.go.kr/knhanes/sub03/sub03_01.do.

## Ethics Statement

The KNHANES 2014 was approved by the Research Ethics Review Committee of the Korea Centers for Disease Control and Prevention. The approval for the KNHANES 2015 was exempted according to Article 2 Paragraph 1 of the Bioethics Act and Article 2 Paragraph 2 Subparagraph 1 of the Enforcement Rules. The participants provided their informed consent prior to the survey and examinations.

## Author Contributions

HL led the overall study, handled and analyzed KNHANES data, and wrote the manuscript. ML contributed to writing discussion section and edited the manuscript. Both authors read and approved the final manuscript.

## Conflict of Interest

The authors declare that the research was conducted in the absence of any commercial or financial relationships that could be construed as a potential conflict of interest.

## Publisher's Note

All claims expressed in this article are solely those of the authors and do not necessarily represent those of their affiliated organizations, or those of the publisher, the editors and the reviewers. Any product that may be evaluated in this article, or claim that may be made by its manufacturer, is not guaranteed or endorsed by the publisher.
